# Heterogeneous effects of socio-economic status on social engagement level among Chinese older adults: evidence from CHARLS 2020

**DOI:** 10.3389/fpubh.2024.1479359

**Published:** 2024-11-29

**Authors:** Wenjia Li, Xinni Zhang, Han Gao, Qinghe Tang

**Affiliations:** ^1^College of Communication and Art Design, University of Shanghai for Science and Technology, Shanghai, China; ^2^Department of Hepatobiliary and Pancreatic Surgery, Shanghai East Hospital, School of Medicine, Tongji University, Shanghai, China

**Keywords:** SES, social engagement, ageing, CHARLS, heterogeneity analysis

## Abstract

**Introduction:**

Older adults benefit from social connections as it aids in their adjustment to the physical and psychological changes that come with aging, thereby improving their health, well-being, and overall quality of life.

**Methods:**

This study utilizes data from the 2020 China Health and Retirement Longitudinal Study (CHARLS) to investigate the influence of Socio-economic Status (SES) on the social activity levels of older persons and the disparities observed among demographic groups, employing the Heckman two-stage analysis and heterogeneity analysis.

**Results:**

The results indicate that SES has a significant positive impact on the social engagement of older adults, and this effect varies across different older groups, with women, married individuals living with a spouse, urban residents, those aged 70–79, and individuals with average health conditions.

**Discussion:**

To effectively address the social needs of older adults, it is essential to prioritize integrating cohesive structural methods that improve social connections. Establishing solid and sustainable social support mechanisms and meeting the social needs of older adults across various SES and demographic groups are crucial in promoting active and healthy aging.

## 1 Introduction

Population aging is a significant phenomenon in social progress and a crucial matter and shared obstacle for all nations globally (1). China is seeing a rapid increase in the proportion of aged people, resulting in a no-table rise in senior citizens (2). China’s aging will deepen in the future, and according to the moderate prediction, China will enter an ultra-elderly society by 2033 (3). Population aging places additional strain on families, society, and economic progress. Therefore, this study aims to examine the impact of SES on social engagement among older adults, and to analyze how this relationship varies across different demographic groups. The primary objective of aging research is to ascertain the elements that contribute to variations among individuals and to mitigate some of the detrimental impacts of aging on cognitive abilities (4). Social participation is crucial in active aging since it significantly influences older individuals’ overall health and well-being. It plays a constructive role in enhancing their health status and subjective well-being. The Activity Theory of Ageing (ATA) contends that older adults keep physically active and healthy as a result of their significant social engagement. Social engagement refers to individuals’ active and proactive integration into their community and social contexts. It involves enhancing personal fulfillment through interaction and sharing resources with others. This method centers on the engagement, communication, and contentment of elderly individuals in communal endeavors. Multiple studies have indicated a strong correlation between the extent of social engagement among elderly individuals and their overall health, quality of life, and mortality rate (5–7). Engaging in social activities positively impacts the physical and mental health, well-being, and overall pleasure with life in older individuals, which are considered important indicators of life contentment (8–13).

Facilitating social engagement and bolstering one’s favorable psychological attributes are crucial in aiding individuals in adjusting to the physiological and cognitive transformations that transpire as they age by cultivating a sense of optimism, revitalizing their outlook on life, and augmenting their psychological welfare (14). In this context, social engagement plays a key role in enhancing overall well-being among older adults. Individuals with a robust sense of self-efficacy have greater confidence in managing interpersonal relationships and achieving social objectives (14). Social communication is crucial in shaping pleasant emotions and is the primary means middle-aged and older persons seek psychological assistance. This support sustains positive emotions and fosters group cohesion (15). Engaging with others within the community or neighborhood is equally important in enhancing one’s sense of well-being and autonomy. Many elderly individuals maintain an active lifestyle and fulfill their social requirements by engaging in volunteer work or participating in informal social gatherings. Meeting social needs is crucial for fulfilling fundamental human requirements, and the absence of such fulfillment can result in adverse mental and physical health outcomes (16). The frequency of conversations with peers in older individuals has a negative correlation with depression. Interacting with friends and neighbors leads to reduced loneliness and improved mental attitude (17, 18). These studies highlight the significance of social activities in the lives of older persons from various physiological and psychological viewpoints, underscoring the collective impact of social contacts on the physical and mental well-being of older adults.

Various factors related to individuals and the environment can either support or impede the extent to which older adults engage in social activities (12). The factors can be classified into four main categories: demographic (such as age and socio-economic status), personal/internal (such as motivation and health), environmental/infrastructural (such as accessibility, transportation, and neighborhood cohesion), and social networks (particularly pre-existing network size) (7). The primary obstacles to physical and social activities among older persons are aging and health state. Additionally, factors such as education level, income, and gender disparities significantly influence the social interactions of older adults (7, 19, 20). Analyzing the factors that impact social involvement in elderly individuals can have a beneficial effect on enhancing their health condition and subjective sense of well-being throughout their later years.

Social connections account for significant differences in subjective health based on SES. In other words, disparities in SES contribute to disparities in health status, making it a significant predictor of many health and disease outcomes (21–24). Positive social contacts are crucial in mitigating stress among socioeconomically disadvantaged populations. Older persons with lower SES are more prone to experiencing worse health, significantly poorer emotional well-being, and shorter life expectancy compared to older adults with higher SES (21, 25–31). The context in which aging takes place significantly influences the extent of aging. Older individuals residing in wealthier areas tend to have much higher health and overall well-being (32). Studies on the involvement of elderly individuals in cultural activities have indicated that a limited percentage of individuals aged 65 and above engage in such activities. Furthermore, the frequency of their engagement in cultural activities is linked to their financial capability to cover expenses (13). Individuals with lower SES often have limited availability of public spaces, and even when such spaces are accessible, they tend to hesitate to utilize them (33). The motivation and obstacles to physical activity differed across older persons of varying SES. Elderly individuals with low SES reported encountering additional obstacles, particularly about their health status, community safety, and understanding of public affairs rules. However, time is a significant obstacle for the high SES group (34). Income-based indicators of SES primarily consider the practical benefits associated with higher SES and may produce contrasting outcomes compared to methods emphasizing education, social class, or relative poverty (22). SES measures a person’s access to resources, their enjoyment of rights, and social standing in society. It directly impacts an individual’s level of involvement in society.

Differences in the impact of SES on older individuals can also be observed across different demographic regions. Individual SES plays a moderating role in the disparities in the quality of physical activity between men and women (35). Older individuals with higher SES tend to experience less height loss (36). Furthermore, SES exhibits a cumulative impact that becomes increasingly noticeable as one age (37). The accumulation hypothesis posits that differences in SES between different groups and the general distribution of health outcomes become more pronounced as individuals age (38). Low educational achievement and low income are the primary demographic factors contributing to ongoing social estrangement, which intensifies with age (39). There is a strong and favorable correlation between higher SES and cognitive ability in older persons. Additionally, social support is essential in mitigating the impact of SES and cognitive performance in an aging population (40, 41). Age discrimination against older adults, deficiencies in social assistance and pension systems, shifts in family dynamics, and the digital divide are other hidden elements contributing to the problem. The presence of social capital, which refers to the resources and connections individuals have for social engagement and overall well-being, along with cultural capital, which relates to the knowledge and skills needed to access social status and opportunities, exacerbates the exclusion and marginalization experienced by older individuals.

Prior research has predominantly examined the physical health aspect, explicitly investigating the correlation between SES and the physical health condition of older individuals. However, studies that explore the psycho-cognitive aspect have primarily focused on cognitive impairments, depression, and similar issues, overlooking the significance of the social aspect of subjective experiences during old age, particularly social capital and social participation. The lives of older individuals are frequently impacted by factors such as social structure and social support. However, there is a dearth of research investigating the variations in social engagement among older persons based on their SES. Within a substantial and comprehensive study like CHARLS 2020, we are afforded the chance to investigate this matter using more extensive and detailed information. The purpose of this study is to examine the impact of SES on the social engagement of older individuals, using data from the CHARLS 2020 survey. Additionally, the study aims to determine how the relationship between SES and social engagement varies across older individuals with varied characteristics. The extent of social participation among older adults indicates their quality of life and health to some degree. Through this study, we aim to gain insight into the dynamics of social life among older adults and empirically investigate the role and influence of SES on their social engagement. This research will provide theoretical guidance and a practical foundation for China’s positive and healthy aging policies.

## 2 Materials and methods

### 2.1 Data source

The data utilized in this paper comes from the China Health and Retirement Longitudinal Study of 2020. CHARLS is a comprehensive interdisciplinary survey funded by The National School of Development and conducted by The Institute of Social Science Survey at Peking University. This extensive project aims to gather high-quality micro-data on households and individuals aged 45 and above in China, explicitly focusing on middle-aged and older adults. The primary objective of CHARLS is to analyze the challenges posed by population aging in China and facilitate interdisciplinary research on aging-related matters. The survey was conducted in 2011 using multistage sampling and population proportionate sampling techniques, with data covering 19,395 samples from 150 districts, 450 villages, and urban community units in 28 provinces around the country. The CHARLS questionnaire encompasses various factors, including demographic characteristics, household composition, health status, utilization of health services, employment status, insurance benefits, household consumption level, and asset status (42). For more detailed and precise information, please visit the official website of CHARLS at^1^. This study utilizes the most recent public data from the 2020 CHARLS Wave 5, formally made available to the public on 16 November 2023. This study focused on individuals aged 60 years and older. After removing samples with missing critical data, a final sample size of 9,533 individuals was selected.

### 2.2 Variable selection

#### 2.2.1 SES

Socio-economic status is primarily determined by education, income, and occupation (43). Given that older adults are predominantly retired and lack autonomous sources of income or have limited earnings, we assess the SES by examining the educational attainment of the participants and the *per capita* household expenditure, as indicated by previous research. *Per capita* expenditure, rather than income, is used to measure household resources because in places such as China, where a great deal of economic activity does not occur through markets, *per capita* expenditure is considered a more reliable measure of household resources and is less prone to measurement errors compared to income (43, 44).

According to the questionnaire’s question, what is your current most significant degree of educational attainment? The level of education has been categorized into four distinct classifications: individuals with no formal education or schooling below primary school, primary school education, secondary education (including junior high school, senior high school, and similar levels), and tertiary education or higher. The *per capita* expenditure was calculated based on the response to the following question in the questionnaire: What is the total monthly expenditure of all household members? The process involves dividing the total household expenditure by the number of household members. Then, *Z*-values are calculated for education and *per capita* expenditure. These values have a mean of zero and a standard deviation of one. The sum of these values is defined as the individual-level SES, with higher values indicating higher individual SES (45).

#### 2.2.2 Social activity level

The dependent variable in this study is social activity level. A social activity index has been created based on the 11 social activities listed in the questionnaire and their frequency. The formula for calculating social activity level is social activity level = social activities multiplied by social frequency (46). Questionnaire item: Have you participated in the following social activities within the last month? What frequency did you engage in the previous option during the last month?

The formula for social activeness is shown in Equation 1:


(1) $$C=\overset{N=9}{\underset{\;i=1}{\sum }}\left(A_{i}*F_{i}\right)$$

It can also be converted to Equation 2:


(2) $$C=A_{1}*F_{1}+A_{2}*F_{2}+\ldots +A_{9}*F_{9}$$

In the formula, C represents the social activity level; A represents the social activity items. According to the questionnaire, nine specific items of social activities are listed, such as hanging out with friends, playing chess and mahjong, and participating in volunteer activities. These items are assigned a value of 0 or 1, indicating whether the social activity has been carried out, respectively. F represents the frequency of each social activity, which is assigned a value ranging from 1 to 3. By multiplying the social activity by frequency, we obtain four possible values: 0, 1, 2, and 3. The calculation involves nine specific social activities, resulting in a final value for the social activity level. The theoretical social activity level ranges from 0 to 27, while the actual social activity level ranges from 0 to 16. Based on the practical requirements of the study, the social activity level of the older adults is categorized as follows: a value of 0 indicates low level, a value between 1 and 3 indicates medium level, and a value of 4 or higher indicates high level.

#### 2.2.3 Covariates

The model included socio-demographic characteristics as covariates, such as age (50–59 = 1, 60–69 = 2, 70–79 = 3), gender (male = 1, female = 2), health status (very good = 1, good = 2, fair = 3, poor = 4, very poor = 5), hukou (rural = 1, urban = 2), and marital status (married and residing with spouse = 1, divorced, widowed, and single = 2).

### 2.3 Statistical analysis

Initially, we conducted descriptive statistics on the sample. Upon examining the variable data, we discovered a significant number of 0-values for social activity level. To avoid the endogeneity problem that this result may cause, this study suggests employing a Heckman two-stage model. It is necessary because the impact of SES on social activity level cannot be accurately measured in a sample of older adults who do not engage in social activities. Additionally, relying solely on a sample of socially active individuals would yield biased effect estimates. The Heckman two-stage model comprises two regression model estimation stages. The first stage involves a binary probit model that utilizes the entire sample to estimate the probability of an older person’s participation in a social activity. This stage aims to address the issue of omitted variables. The second stage employs Ordinary least squares (OLS) regression to estimate the level of social activity among older individuals. Subsequently, we performed heterogeneity analyses for demographic variables to investigate the impact of SES on the social engagement of several groups of elderly individuals. This study used SPSS 26 for database merging and sample screening and STATA 17 for data analysis.

## 3 Results

### 3.1 Descriptive statistics

Table 1 presents descriptive statistics and information on the variables. It shows that the ratio of male to female participants in this study was roughly similar, with 48.8% male and 51.2% female. Additionally, the average age of the participants was 68.6. China has a significant rural population, resulting in a substantial difference in urban and rural families. Specifically, 43.6 percent of these households have no formal education or completed less than primary schooling. Approximately 54.9% of the participants reported having an average level of self-health, and a significant 55.9% had poor social engagement, indicating that over half did not engage in any social activities in the previous month. The *per capita* expenditure varied substantially, ranging from 0 to 50,000. The primary independent variable, SES, ranged from −2 to 45.1, with higher values indicating a higher personal SES.

**Table 1:** Descriptive statistics of all variables (*n* = 9,533).

Variables	Frequency	Percentage	Mean (SD)	Min	Max
Gender					
Male	4,648	48.8			
Female	4,885	51.2			
Hukou					
Rural	6,977	73.2			
Urban	2,556	26.8			
Age			68.6 (6.4)	60	87
60–69	5,959	62.5			
70–79	2,845	29.8			
80–89	729	7.6			
Marital status					
Married	6,955	73.0			
Single/Widowed/Divorced	2,578	27.0			
Health status					
Very good	845	8.9			
Good	969	10.2			
Fair	5,234	54.9			
Poor	1840	19.3			
Very poor	645	6.8			
Education					
Below primary	4,158	43.6			
Primary	2099	22.0			
Secondary	3,084	32.4			
Tertiary or higher	192	2.0			
Social activity levels			3.24 (2.2)	0	16
Low	5,327	55.9			
Middle	2,967	31.1			
High	1,239	13.0			
*Per capita* expenditure			761.6 (1073.2)	0	50,000
SES			0 (1.4)	-2	45.1

### 3.2 Heckman two-stage model

The Heckman two-stage model is an appropriate approach for addressing the issue of endogeneity resulting from sample selection bias. It is a widely employed regression model and method in empirical quantitative research. Given that the decision to participate in social activities can be classified as a yes or no choice, a binary probit model is used to determine whether or not older adults engage in social activities. The independent variable in this model is an external factor that influences the social activities of older adults. In addition, an Inverse Mills Ratio (IMR) is estimated for each sample, which is used to correct for sample selection bias.

The probit choice model used to analyze the engagement of older individuals in social activities is shown in Equation 3.


(3) $$P\left(y=1|X\right)=\emptyset \left(\alpha +\beta_{i}\overset{n}{\underset{i=1}{\sum }}x_{i}\right)$$

In Equation 3, y represents the binary decision variable indicating whether an older individual engages in social activities. $x_{i}$ is the column vector of the variable matrix X, and *α* and *β* are the parameters that need to be estimated.

The IMR is derived from the estimation results of the probit model and is denoted as μ in Equation 4.


(4) $$\mu =\frac{\varphi \left(\alpha +\beta_{i}\sum_{i=1}^{n}x_{i}\right)}{\emptyset \left(\alpha +\beta_{i}\sum_{i=1}^{n}x_{i}\right)}$$

In the second stage of the model, OLS regression was used to estimate the social activity level of older individuals. This stage involved including IMR as a correction factor and other variables from the original regression model and estimating the regression parameters. If the IMR parameter, which serves as the correction term in the second stage, lacks significance, it indicates the absence of selective bias in the initial regression equation. In such cases, it can be directly estimated using OLS. Conversely, if the IMR parameter is significant, it suggests the presence of sample selective bias, which should be addressed by employing the Heckman two-stage model for correction.

Substituting IMR into the OLS regression equation for social activity level of older adults, we derived the second-stage equation that represents the level of social engagement among older adults.


(5) $$C_{i}=\delta_{0}+\delta_{i}\overset{n}{\underset{i=1}{\sum }}Z_{i}+\omega \mu +\epsilon_{i}$$

In Equation 5, ∁ represents the social activity level, $Z_{i}$ represents the independent variable that influences social activity level, and $Z_{i}$ is a subset of the variable matrix X. This means that there is at least one variable that affects the choices made by older adults in terms of participation, but this variable does not have a biased effect on social activity level. The waiting estimate parameters of the independent variables are represented by $\delta_{i}$ and ω . The intercept term is denoted as $\delta_{0}$ , and the random disturbance term is represented by $\epsilon_{i}$ .

As shown by Table 2, the IMR value obtained from the analysis of the Heckman two-stage model is −2.875, which is statistically significant at the 1% level, indicating the presence of selective bias in the sample. Consequently, the decision to use the Heckman two-stage model in this study is reasonable. The Heckman two-stage model reveals that the first-stage regression results indicate the impact of various factors and variables on the participation of older adults in social activities. The analysis demonstrates that apart from marital status, SES, gender differences, self-rated health, age, and hukou significantly affect the involvement of older adults in social activities, with varying degrees of statistical significance. The second phase of the two-stage Heckman model focuses exclusively on a subset of elderly individuals who engage in social activities. The study used model-fitting to determine the factors influencing older adults’ social engagement. The variables considered were SES, gender differences, self-rated health status, age, marital status, and hukou. It is found that all of these variables have a significant impact on social activity level.

**Table 2:** Heckman two-stage model.

Variables	Model 1		Model 2	
	Coefficient	Std. err.	Coefficient	Std. err.
SES	0.042***	0.012	0.056**	0.024
Health	−0.054***	0.014	−0.094***	0.035
Gender	0.071***	0.026	−0.335***	0.035
Age	−0.013***	0.002	−0.061***	0.006
Marriage	0.046	0.030	−0.160**	0.078
Hukou	0.291***	0.030	0.423***	0.072
IMR	-	-	−2.875***	0.393
Cons	0.371	0.153	7.863	0.403
R-squared	0.052			
Observation	9,533		4,206	

*p<0.1; **p<0.05; ***p<0.01.

### 3.3 Heterogeneity analysis

#### 3.3.1 Gender heterogeneity

The heterogeneity analysis in Table 3 reveals that the SES coefficient for males is 0.034, indicating no significant association with social activity level. On the other hand, the SES coefficient for females is 0.078 and *p*<0.05, indicating a highly significant impact on social activity level.

**Table 3:** Gender heterogeneity analysis.

Variables	Male	Female
SES	0.034 (0.943)	0.078** (2.411)
Hukou	0.327*** (3.029)	0.527*** (5.417)
Marriage	−0.243* (−1.854)	−0.140 (−1.475)
Age	−0.073*** (−8.629)	−0.050*** (−6.841)
Health	−0.129** (−2.349)	−0.059 (−1.304)
*r* ^2^	0.047	0.043

*p<0.1; **p<0.05; ***p<0.01.

Therefore, the impact of women’s SES on social activity level is more substantial than men’s. This observation can be somewhat attributed to variations in social roles and expectations, and it also indicates the distinct preferences men and women have in social relationships. Historically, males have traditionally assumed greater responsibility for managing family money and dedicate more time to their professional endeavors than to engaging in regular social activities. On the other hand, society typically assigns women more significant social responsibilities, such as becoming event coordinators within the family and community. This distinction in social roles may persist throughout their later years (47). Men and women exhibit distinct preferences and behaviors in social interactions. Older women, in particular, are more prone to being socially supportive and emotionally engaged, and they are more inclined to cultivate deeper ties, creating denser and more stable social networks. Women experience a notably superior standard of living in social interactions (48). In contrast, older men may prioritize the practical and leisurely components of social events, which explains their comparatively lower participation rates.

#### 3.3.2 Marital status heterogeneity

The heterogeneity analysis in Table 4 reveals that the SES index for older individuals who were married and living with their spouses is 0.066, *p*<0.05, indicating substantial statistical significance. On the other hand, the SES index for older adults who are single, divorced, or widowed is 0.033 and does not demonstrate any statistical significance.

**Table 4:** Marital status heterogeneity analysis.

Variables	Married	Single/Widowed/Divorced
SES	0.066** (2.215)	0.033 (0.822)
Gender	−0.373*** (−4.609)	−0.222* (−1.826)
Hukou	0.434*** (5.004)	0.382*** (2.934)
Age	−0.063*** (−8.613)	−0.060*** (−7.248)
Health	−0.115*** (−2.690)	−0.037 (−0.596)
*r* ^2^	0.042	0.054

*p<0.1; **p<0.05; ***p<0.01.

The SES of older persons who are married and cohabiting with their spouses tends to have a more significant impact on their level of social engagement. Marital relationships typically involve a higher level of emotional interaction and fulfillment, allowing couples to share resources and experiences and provide emotional support to one another. This consistent emotional support and companionship can positively affect on both physical and mental well-being (49, 50). Increased levels of mental health and well-being can result in heightened drive and self-assurance, which can encourage increased social engagement. Married couples with a high SES possess more excellent social resources, including a more extensive social network, broader social interactions, and increased social capital (51). These resources contribute to their enhanced social engagement. The marriage partnership typically entails specific societal tasks and obligations, including providing financial support for the family and ensuring the raising and education of children (52, 53). Married couples with a high SES are more inclined to assume more significant social duties. They also prioritize the maintenance and growth of their social connections to fulfill the expectations of their families and society (54). On the other hand, older individuals who are divorced, widowed, or single may have more tremendous obstacles in their social lives because they lack consistent emotional support and a clear social role identity. Consequently, they will demonstrate lower levels of social engagement due to the combined influence of these factors.

#### 3.3.3 Hukou heterogeneity

Regarding the hukou type, Table 5 reveals that the SES coefficient for rural senior individuals is 0.005, and the impact on social activity level is insignificant. Conversely, the SES coefficient for urban elderly individuals is 0.134, *p*<0.01, indicating a significant effect on social activity level.

**Table 5:** Hukou heterogeneity analysis.

Variables	Married	Single/Widowed/Divorced
SES	0.005 (0.184)	0.134*** (2.976)
Gender	−0.421*** (−5.655)	−0.162 (−1.157)
Marriage	−0.130 (−1.547)	−0.254 (−1.514)
Age	−0.058*** (−9.430)	−0.068*** (−5.983)
Health	−0.063* (−1.662)	−0.180** (−2.298)
*r* ^2^	0.049	0.039

*p<0.1; **p<0.05; ***p<0.01.

The impact of SES on social engagement is more pronounced among older persons living in urban areas than those living in rural areas, which is inseparable from the superior social resources and social environment found in urban regions (55). Elderly individuals residing in urban areas typically participate in diverse social activities and have access to multiple opportunities for social connection, such as social groups, recreational facilities, and cultural venues. They benefit from improved transportation, communication, and city infrastructure, which inadvertently enhances their motivation to venture outside (56). Residing in an urban typically entails heightened competition, with individuals being more susceptible to social pressure and expectations to uphold social connections and engage in communal endeavors. Consequently, older persons living in cities may prioritize participating in social activities, and they may be more motivated to enhance their SES to acquire additional social resources and recognition. Conversely, in rural regions, elderly individuals face limited options and possibilities for socializing due to a relative lack of resources or inconvenient transportation (57). As a result, the range and frequency of their social activities may be restricted. As urbanization speeds up, the social environment and distribution of resources between urban and rural areas are undergoing subtle changes (58, 59). It is essential to establish an inclusive and supportive environment for the social engagement of older individuals through urban planning and community development.

#### 3.3.4 Age heterogeneity

Table 6 reveals that the coefficients of SES for older adults in the age groups 60–69 and 80–89 are 0.016 and 0.106. These coefficients do not demonstrate a significant impact on social activity level. However, the coefficient of SES for older adults in the 70–79 age group is 0.135, *p*<0.01, indicating high significance, exhibiting the strongest association between SES and social activity level.

**Table 6:** Age heterogeneity analysis.

Variables	60–69	70–79	80–89
SES	0.016 (0.496)	0.135*** (3.214)	0.106 (1.422)
Gender	−0.482*** (−5.591)	0.135*** (3.214)	−0.146 (−0.734)
Hukou	0.432*** (4.602)	0.415*** (3.145)	0.228 (1.127)
Marriage	−0.216** (−2.053)	−0.251* (−1.877)	−0.116 (−0.573)
Health	−0.129*** (−2.886)	−0.037 (−0.565)	−0.090 (−0.785)
*r* ^2^	0.026	0.024	0.005

*p<0.1; **p<0.05; ***p<0.01.

We speculate that individuals between the ages of 60–69 may need time to adjust their lifestyle and social connections after retirement, and during this adjustment period, SES seems to have less influence on their social activism compared to older age groups. However, among individuals aged 70–79, who are mostly experiencing a time of stability in terms of health and energy, the influence of SES on the frequency of social engagement is at its highest level. Those with high SES have a more remarkable ability to participate actively in social activities, which helps them retain social connections and promotes their physical and mental well-being. SES has a cumulative impact (37), meaning those with higher SES are more likely to fulfill their social demands. While individuals between the ages of 80 and 89 may experience health issues that limit their engagement in social activities, the primary obstacle to physical exercise in old age is the process of aging itself (20). As individuals age, they typically undergo a decrease in physiological functioning, which includes reductions in physical mobility, perceptual ability, and immune function (60). The physiological changes experienced by older individuals can make them more prone to weariness and discomfort, leading to decreased motivation and capacity to participate in social activities, ultimately limiting the frequency and range of their involvement. The physiological changes in individuals can lead to psychological changes that may impact their interest in social activities. Older persons are particularly susceptible to experiencing anxiety, loneliness, and depression as a result of these changes (61, 62), which can further decrease their participation in social activities. Furthermore, the social networks of elderly individuals often transform, resulting in a reduction in the size of their social circles due to the natural aging process and the loss or relocation of friends and family (63, 64).

#### 3.3.5 Health status heterogeneity

The analysis of health status categorized into five levels in Table 7 shows that the SES index of elderly individuals with a fair health status is 0.105 with *p*<0.01, which indicates a highly significant association with social activity level. On the other hand, the SES index of elderly individuals with the other four health statuses, −0.014, 0.044, −0.045, and 0.041, does not show a significant relationship with social activity level. The impact of SES on social activity level was most substantial among older persons who reported having a fair health state.

**Table 7:** Health status heterogeneity analysis.

Variables	Very good	Good	Fair	Poor	Very poor
SES	−0.014 (−0.163)	0.044 (0.503)	0.105*** (3.058)	−0.045 (−0.815)	0.041 (0.784)
Gender	−0.539** (−2.278)	−0.340 (−1.537)	−0.307*** (−3.340)	−0.365** (−2.495)	−0.158 (−0.668)
Hukou	0.380 (1.470)	0.741*** (3.258)	0.398*** (4.133)	0.393** (2.331)	−0.044 (−0.156)
Age	−0.070*** (−3.354)	−0.100*** (−5.667)	−0.057*** (−7.726)	−0.043*** (−3.451)	−0.072*** (−3.260)
Marriage	−0.185 (−0.642)	−0.211 (−0.846)	−0.206* (−1.928)	−0.029 (−0.179)	−0.130 (−0.517)
*r* ^2^	0.043	0.094	0.048	0.025	0.036

*p<0.1; **p<0.05; ***p<0.01.

Older individuals in good or excellent physical condition, although having a poor SES, may fulfill their socialization requirements through alternative methods, such as receiving assistance from their family or engaging in outdoor activities (65, 66). Conversely, older persons with lower health status may face limitations in their capacity to engage fully in social activities despite having a superior SES (67). The influence of SES may have a limited effect on both cohorts of elderly individuals. However, older individuals in good overall health, who have some health issues but are not significantly impaired by them, and who prioritize self-care (68), are more susceptible to the influence of SES. A higher SES can offer them more excellent resources and assistance in managing their health problems while providing chances to participate in social activities. In this scenario, the impact of SES on the frequency of social activity is expected to be the most notable, as it directly influences individuals’ capacity and chances to participate in social activities.

## 4 Discussion

Research has shown that the social engagement of older individuals is affected by their SES and various other characteristics. It emphasized that consistently engaging in regular and meaningful social activities is crucial for older persons, as it is linked to notable improvements in their health, well-being, and cognitive abilities (7). Our findings support prior studies indicating that personal, environmental, and social factors influence social involvement in elderly individuals (6, 12). The primary independent variable in this study is SES, which encompasses the educational attainment and *per capita* expenditures of older adults. The study found that SES had a significant positive impact on the social activity level of older adults. Specifically, older adults with higher SES exhibited higher social activity levels. Higher SES typically indicates a greater abundance of social resources and connections. Older adults with high SES have more financial resources to engage in various social activities (20). They also have easier access to social resources and support, allowing them to establish and maintain social relationships. Additionally, individuals with high SES tend to enjoy higher social status and recognition in society. Conversely, elderly individuals with low SES may experience financial, occupational, and familial caring demands that lead to reduced opportunities for social interaction. Elderly individuals who have their socialization requirements fulfilled experience a corresponding enhancement in their quality of life, resulting in beneficial effects on their physical and mental well-being (8, 69).

Simultaneously, we discovered that the influence of SES on social engagement differs among older individuals. Regarding gender disparities, the more significant impact of SES on women compared to males may be attributed to societal expectations regarding women’s social duties. On the other hand, older individuals who are married and cohabiting with their spouses are more susceptible to the influence of SES, which could be attributed to their robust familial connections and social networks. The fact that the social activity level of urban older adults is more affected by SES highlights the unequal allocation of social resources and opportunities. The primary influence of SES on social engagement among those aged 70–79 may be attributed to life changes and social expectations within this age range. The fact that older adults with “fair” health status are more affected by SES may reflect the moderating effect of health status on social activity level. These findings offer valuable insights into the factors that influence the social behaviors of older adults and the provision of social support and interventions for them. And suggests that when developing social policies for older adults, it is crucial to consider the characteristics and variations in SES among individuals (70). Furthermore, efforts should be directed toward promoting social engagement among older adults to enhance their overall quality of life.

The extent to which society addresses the issue of social involvement among older individuals is demonstrated by its complete grasp of and reaction to their social demands in the long term. Comprehending the aging patterns and acknowledging the significance of the social aspect of the status of older individuals with varying SES, it is crucial to recognize that as the older population increases, social structures, family dynamics, and community support systems are likely to change. Furthermore, disparities in SES play a crucial role in driving inequalities in allocating social support and resources. Poverty and inequality significantly impede the social cohesion of minority participant groups. Additionally, the evolving social interaction needs of older individuals across different life stages and environments, as well as the ability of social systems to adapt to these changes and provide appropriate support and services, necessitate the implementation of effective, sustained, and specifically tailored interventions. To promote active and healthy aging among older individuals across different SES, it is imperative to establish a sustainable support mechanism. This can be achieved by considering various factors, such as public policies and social services, which will contribute to fulfilling their social needs and developing a robust social support system.

## 5 Conclusion

This study, utilizing data from CHARLS 2020, systematically explores the impact of SES on the social engagement of older adults and examines the variations across different subgroups. The findings indicate that SES has a significant positive effect on the social activity levels of the elderly, with notable differences across gender, marital status, residential area, age, and health status. Specifically, the influence of SES is more pronounced among women, married individuals living with their spouse, urban residents, those aged 70–79, and individuals in fair health. This highlights that these groups are more likely to be either constrained or facilitated by social resources and economic conditions, underscoring the need for tailored social support strategies to address the unique needs of different SES groups. By elucidating the interaction between specific social engagement patterns and socioeconomic backgrounds, this study provides new theoretical foundations for developing and implementing more targeted social services and policies for the elderly.

At a broader level, SES inequality has the potential to exacerbate social isolation and health disparities as aging and urbanization processes unfold. This underscores the necessity of transitioning from a narrow focus on resource allocation to a more comprehensive approach that supports diverse models of social participation. From both policy and practical standpoints, future initiatives for elderly social activities should prioritize enhancing the depth and cohesion of social interactions through structural measures, with a particular focus on providing greater social support and resources for low-SES groups. This would involve establishing a sustainable social support system, designing differentiated social strategies, and promoting multi-level, cross-sector platforms for social participation. By leveraging technology and innovative social platforms, policymakers can address the barriers faced by low-SES older adults in engaging in social activities. This would help mitigate health disparities driven by socioeconomic inequalities, promote cross-class social integration and health equity, and better meet the social needs of diverse SES groups. Ultimately, such efforts would enable older adults to better manage the physical and psychological challenges of aging, fostering positive and healthy aging outcomes.

This study elucidates the determinants of social engagement among older adults and highlights the disparities among various groups of older adults. However, it is essential to acknowledge the presence of several drawbacks and constraints in this study. Firstly, the study relies on cross-sectional data for analysis and does not include long-term tracking observation to uncover the temporal pattern of social activity level in older individuals or establish a causal relationship between the influencing factors. Furthermore, the study primarily focused on SES and demographic-geographic factors. However, it is essential to note that due to the data limitations of CHARLS, certain potential confounding variables, such as cultural background and residential environment, were not considered. These factors could potentially have a significant influence on the social engagement of older adults. Ultimately, the study mainly employed quantitative analysis methods to examine specific statistical associations. However, it is crucial to note that quantitative analysis may not comprehensively capture and elucidate intricate social phenomena, such as social behaviors and psychological states. In the future, we will conduct a prospective cohort study to empirically research the social engagement of older adults in different groups. We will examine various factors that affect their social engagement, focusing on variables such as cultural background and living environment. The forthcoming investigations are expected to substantially improve the social engagement and well-being of elderly individuals.

## Data Availability

The datasets presented in this study can be found in online repositories. The names of the repository/repositories and accession number(s) can be found at: https://charls.pku.edu.cn/en/ (accessed on 21 May 2024). Further inquiries can be directed to the corresponding author.

## Footnotes

^1^ https://charls.pku.edu.cn/
